# A rare case of invasive pulmonary aspergillosis presenting as organizing pneumonia due to *Aspergillus niger* in an immunocompetent host

**DOI:** 10.1016/j.rmcr.2021.101503

**Published:** 2021-08-25

**Authors:** Raphaël Schils, Antoine Altdorfer, Filip Moerman, Thierry Weber, Michèle De Waele, Johan Maertens, Benoit F. Pirotte

**Affiliations:** a Department of Internal Medicine and Infectious Diseases, Liège University Hospital, 4000, Liège, Belgium; b Department of Internal Medicine and Infectious Diseases, CHR de la Citadelle, 4000, Liège, Belgium; c Department of Pneumology, CHR de la Citadelle, 4000, Liège, Belgium; d Department of Cardiothoracic Surgery, CHR de la Citadelle, 4000, Liège, Belgium; e Department of Hematology, University Hospital Leuven, 3000, Leuven, Belgium

**Keywords:** Invasive pulmonary aspergillosis, Organizing pneumonia, *Aspergillus niger*

## Abstract

Invasive pulmonary aspergillosis associated with organizing pneumonia is increasingly described and mainly affects the immunocompromised individual. Most of Aspergillus invasive infections in both immunocompetent and immunocompromised patients are attributed to Aspergillus fumigatus. Herein we describe a clinical case of pulmonary *Aspergillus niger* infection presenting as an organizing pneumonia in an immunocompetent patient. A wedge resection and two successive cures of azoles (voriconazole 6 weeks then itraconazole 6 weeks) were necessary for the patient to be totally recovered. The association of OP and IPA is rare. The involvement of *A. niger* makes it even rarer, this is the reason why we decided to report on this case.

## 1 Introduction

Organizing pneumonia (OP) is a clinical, radiological and histological entity corresponding to a lung tissue repair process. It can either be secondary to a pulmonary lesion (infection, toxicity, radiotherapy, …) or associated with a primary pulmonary pathology (vasculitis, neoplasia, pulmonary infarction, …). In rare cases, it is considered idiopathic and is classified as cryptogenic organizing pneumonia [1]. *Aspergillus niger* is a fungus widely found in the environment and especially in the molds of many fruits and vegetables. Invasive pulmonary aspergillosis (IPA) associated with OP in the immunocompetent patient is not frequently described. Here we report a rare case of invasive pulmonary aspergillosis presenting as organizing pneumonia due to *A. niger*.

## 2 Case report

A 27-year-old man was admitted to our emergency department complaining about cough, recurrent episodes of hemoptysis and B symptoms (weight loss and nocturnal sweating) that had persisted for several weeks and coming to a crescendo. Upon presentation, physical examination was strictly. The patient was free of medical issue, recent corticotherapy and addiction. He had been living in Belgium for six months and previously lived many years in Spain and Morocco.

**Fig. 1:**
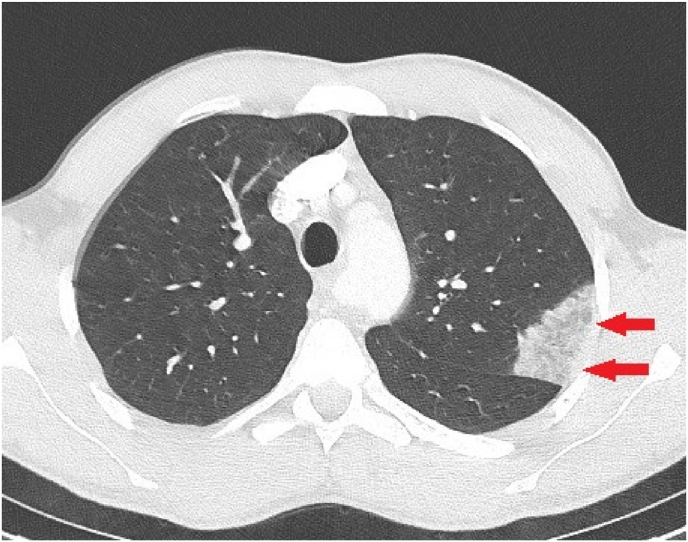
Chest CT showing the condensation in the left upper lobe (red arrows). The bilateral ground glass opacities are not present on this section. (For interpretation of the references to colour in this figure legend, the reader is referred to the Web version of this article.)

Basic investigations were subsequently performed in the emergency unit at day 0. The results of the laboratory tests disclosed that the eosinophil count was significantly high (1.46 × 10³/mm³). C-Reactive Protein, white cells count and d-dimers were normal. A lung CT scan was performed and showed bilateral apical ground-glass opacities associated to an atypical condensation in the left upper lobe apical segment (Fig. 1). The patient was then hospitalized and underwent bronchoalveolar lavage (BAL) at day +1. Cultures, Ziehl-Neelsen staining for mycobacteria and PCR *Mycobacterium tuberculosis* were negative.

**Fig. 2:**
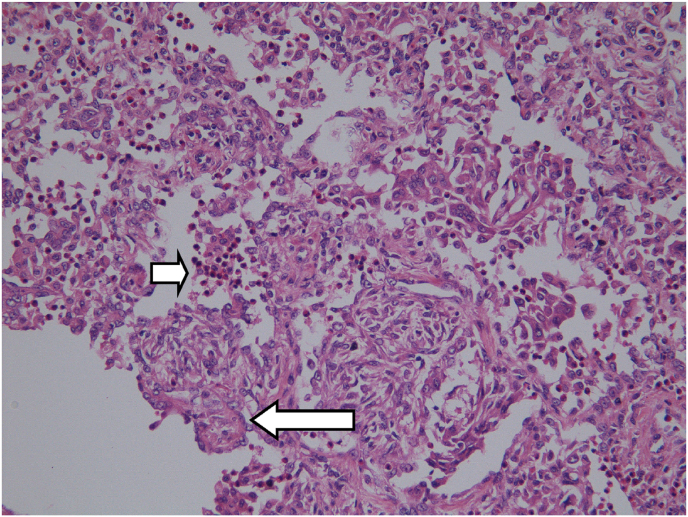
Histological section showing foci of pneumonia with many eosinophilic polynuclear cells (small arrow). Some alveolar ducts and alveoli are the site of endoluminal obstruction by fibrous granulation tissue consisting of inflammatory cells, fibroblasts, and connective tissue (big arrow). Magnification ×400. Haematoxylin and eosin stain.

In view of the persistent and worsening hemoptysis, patient underwent thoracoscopic wedge resection of the concerned left upper lobe at day +3. The thoracoscopic exploration was marked by a blackish indurated mass on the left upper lobe which was therefore resected. Pathologic examination revealed the presence of foci of pneumonia with numerous eosinophilic polynuclear cells (Fig. 2).

The following days in our department were marked by complete resolution of symptoms without benefit of any treatment. A thorough etiological laboratory test was performed at day +5, such as auto-immune tests (anti-nuclear antibodies and anti-neutrophil cytoplasmic antibodies), complement system markers (C3, C4 and CH50), protein electrophoresis, HIV serology, lymphocytic count (CD4+/CD8+) and immunoglobulins (IgM, IgA and IgG). No abnormalities were found but lingered a persistent hypereosinophilia. Interferon-gamma release assays (IGRA) and direct examination on sputum smear were negative. Serum galactomannan optical density index was also negative.

Anatomopathological examination showed features consistent with a bronchiolitis obliterans organizing pneumonitis and marked hypereosinophilia. No branching hyphae were observed. A PET-CT was performed at day +8 and displayed moderately hypermetabolic changes at the apex of the left lung (place of wedge resection) but no other hypermetabolic lesion. A corticosteroid therapy based on methylprenisolone 64mg was started at day +7.

**Fig. 3:**
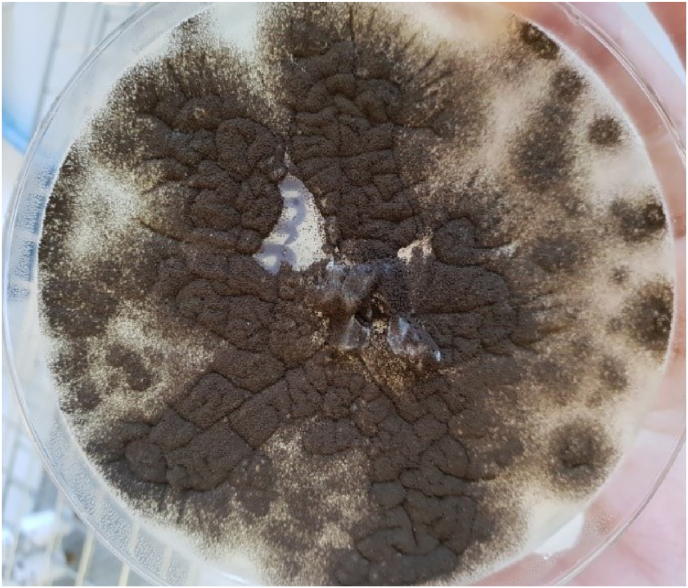
Aspergillus niger growing on Sabouraud dextrose agar. Aspergillus niger is the only pathogenic Aspergillus whose head is radiated, biserial and black at maturity.

As the follow-up was completed and the patient relieved from his symptoms, he was discharged 10 days after admission while tapering off the oral corticotherapy. However, a few days later, microbiology culture of the lung sample revealed black colonies with a granular appearance whose microscopic characteristics were reminiscent of *Aspergillus niger* (Fig. 3) leading us to consider the diagnosis of IPA. PCR Aspergillus on the lung sample was positive with a low cycle threshold (17 cycles). Corticotherapy was immediately stopped and the patient went on oral Voriconazole 200mg twice daily, without loading dose, for 6 weeks.

Compliance and tolerance were excellent. No adverse effect was encountered during those 6 weeks. However, a week after treatment withdrawal, the patient noticed the quick resurgence of the same symptoms that had initially brought him to the emergency room (especially hemoptysis and asthenia). By performing a chest scan, we highlighted the presence of a consolidation at the operative site. The patient therefore benefited from a treatment by itraconazole 200mg twice daily for another 6 weeks. The evolution was quickly spectacular and there have never been any new relapses to this day (2 years follow-up). The indoor environment analysis service was asked to inspect the patient's workplace and home but no Aspergillus was found.

## 3 Discussion

Most of Aspergillus invasive infections in both immunocompetent and immunocompromised patients are attributed to Aspergillus fumigatus. The second and third most common agents are *A. flavus* and *A. terreus*. As a result, there are very few reported cases of immunocompetent individuals infected by *A. niger* whether the pathology is localized or disseminated [[2], [3], [4]]. This is partly explained by the fact that *A. niger* is a low virulence fungus particularly because of its large size conidia and strong cell wall junctions making lower airway penetration laborious [5]. In vitro, *A. niger* seems to have the smallest rate of invasive disease among the Aspergillus species in patients with hematologic malignancies [6]. Steinbach and al. showed that the rate of invasive infection due to *A. niger* was relatively low in both immunocompromised and immunocompetent patients (8.7% out of 960 patients compared to 72% for A. fumigatus) [7].

The association of OP and IPA is rare. The involvement of *A. niger* makes it even rarer. Therefore, the clinical presentation has been confused with cryptogenic organizing pneumonia (COP), leading to inadequate treatment with steroids only. Considering that the patient was free of any previous medical issue, we could not predict his respiratory system being vulnerable to aspergillosis invasion.

Voriconazole is the cornerstone of initial therapy of IPA as recommended by Infectious Diseases Society of America (IDSA), the European Society for Clinical Microbiology and Infectious Diseases (ESCMID) and the European Respiratory Society (ERS) in 2018 [8,9]. Anti-fungal treatment should be continued for 6–12 weeks depending on the patient's evolution and co-morbidities such as level of immunosuppression [7]. The patient was therefore treated with voriconazole 200mg twice daily for 6 weeks, without corticotherapy, during which the evolution was favorable. Nevertheless, the relapse of symptoms having been considered an early recurrence, itraconazole was started as salvage therapy even if this triazole isn't associated with the best results [9]. However it remains a valid option for salvage therapy [9]. No secondary prophylaxis was prescribed due to absence of underlying immunosuppression and good outcome after second cure.

In the case of OP, management depends entirely on the etiology identified. To this day, there are no specific guidelines on the management of OP with IPA, in particular on the value of prescribing corticosteroids in combination with antifungals. However, some authors recommend this combination [10].

There are only a few case reports which are similar to the present case. Recently, without undergoing surgery, a 62-year-old immunocompetent man survived from co-occurrence of IPA and OP [10]. In 2017, a patient suffered from OP in the aftermath of chronic pulmonary aspergillosis but was treated with success after undergoing surgery and corticotherapy [11]. In 2018, a man was diagnosed with the same pathology after benefiting mammalian target of rapamycin (mTOR) inhibitor therapy [12]. Another case report, in 2014, describes specifically the scarce association of OP and IPA in a immunocompetent patient but yet doesn't involve *A. niger* [13].

This case emphasizes the insidious presentation of aspergillosis. Any delay in diagnostic and therapeutic management can be lethal. To the best of our knowledge, this is the first European case of *A. niger* IPA with OP successfully treated with surgery and antifungal therapy.

## Funding

This manuscript did not receive any specific grant from funding agencies in the public, commercial, or not-for-profit sectors.

## Declaration of competing interest

None.
